# Physical exercise among radiologists in Saudi Arabia: a cross-sectional study

**DOI:** 10.1186/s13690-020-00450-x

**Published:** 2020-08-11

**Authors:** Mohammed Al Gadeeb, Ali Hassan, Omran Al Dandan, Malak Al Shammari, Mawaheb Kalalah, Najwa Zabeeri, Abdulaziz Farea, Danya Gari, Hind S. Alsaif

**Affiliations:** 1 Department of Radiology, King Fahd Hospital of the University, Imam Abdulrahman Bin Faisal University, Al-Khobar, Saudi Arabia; 2 Department of Family and Community Medicine, College of Medicine, Imam Abdulrahman Bin Faisal University, Dammam, Saudi Arabia

**Keywords:** Physical exercises, Radiologist, Musculoskeletal symptoms

## Abstract

**Background:**

The practice of clinical radiology has become more sedentary in the era of the Picture Archiving and Communication System. Physical inactivity is a well-known risk factor for various chronic diseases. This study aimed to determine the frequency and pattern of physical exercises among radiologists in the Eastern Province of Saudi Arabia and the association between physical exercises and the prevalence of work-related musculoskeletal symptoms.

**Methods:**

An online survey was sent to radiologists in all hospitals (academic, public, and private) in the major cities of the Eastern Province of Saudi Arabia. It covered information about demographic characteristics and the frequency and pattern of physical exercises. It also included an evaluation of work-related musculoskeletal symptoms using the Nordic Musculoskeletal Questionnaire. This survey of 263 radiologists was conducted in April 2019. The study outcome was the presence of disabling musculoskeletal symptoms in any body region which restricted the performance of normal activities within the last 12 months. The study results were analyzed descriptively using the Chi-square test.

**Results:**

The survey was completed by 198 participants (111 men and 87 women) with a response rate of 75.3%. Most participants (71.2%) were less than 40 years. Eighty-three men (74.8%) did a physical exercise at least weekly, compared to 45 (51.7%) women. Men were more likely to engage in various physical exercises than women. Overall, 60.9% of participants who did not do any physical exercise regularly (less than monthly) reported having disabling neck pain. This figure was found lower among participants who did physical exercises monthly (45.8%) or at least weekly (32.8%). A similar pattern was observed with shoulder pain, with 45.7% found in participants who did not exercise and only 25.8% in those engaging in physical activities at least weekly.

**Conclusions:**

Physical inactivity is common among radiologists, especially female ones, in the Eastern Province of Saudi Arabia. The physical inactivity was significantly associated with work-related musculoskeletal symptoms. Gender-specific health promotion programs are needed to mitigate the negative health outcomes due to the sedentary nature of the radiology current practice.

## Background

Innovations in science and technology have affected all aspects of modern life and have remarkably transformed several fields of work. However, these developments have also contributed to a significant increase in sedentary behaviors across fields, and medicine is no exception to this pervasive trend.

In the last few decades, clinical radiology has evolved dramatically owing to the introduction of electronic medical records and the picture archiving and communication system (PACS). The transition from hard-copy film to the PACS has enabled clinicians to readily access radiological images and reports, at any time or place. While this development has increased productivity and efficiency [1, 2], it also has some inherent drawbacks. Today, radiologists spend long hours in front of computer screens to examine and analyze medical images, which increases sedentary behavior and physical inactivity.

There is strong evidence that regular physical exercise has positive effects on health. These include the prevention and management of cardiovascular diseases, diabetes mellitus, obesity, and certain cancers [3–5]. In contrast, physical inactivity — which is prevalent worldwide and is observed in approximately 20% of all adults — is associated with a wide range of negative health outcomes, such as increased mortality [6, 7]. Prolonged sitting time, in particular, is an independent risk factor for mortality. A meta-analysis conducted in 2016, involving over 1 million individuals, showed that a daily sitting time of over 8 h is associated with increased all-cause mortality [8]. Interestingly, however, other studies revealed that this risk was attenuated among individuals engaging in physical exercises [9, 10].

Musculoskeletal symptoms are common worldwide [11]. Musculoskeletal complaints, similar to physical inactivity, are associated with several negative determinants of health [12, 13]. Previous cross-sectional studies examining the relationship between sedentary behaviors and musculoskeletal symptoms have identified associations between physical inactivity and a higher prevalence of musculoskeletal symptoms [14–17].

Sedentary behavior has increased among radiologists since the advent of the PACS [18], putting this group at potential risk of musculoskeletal complaints. Thus, our study aimed to investigate the prevalence of physical inactivity among radiologists practicing in the Eastern Province of Saudi Arabia and to examine the association between the frequency of physical exercises and work-related musculoskeletal symptoms.

## Methods

### Study design

The survey was designed using the QuestionPro survey software (Seattle, WA, USA). In our study, we used an online survey format because it is easily accessible, time-saving, and cost-effective. Besides, the survey was designed to be taken anonymously — without personal identification data requested or stored — and lasted within approximately 7 min.

A cover letter was provided along with the survey question. It stated the purpose of the study, informed participants about the voluntary nature of their participation, and assured their anonymity. Participants were encouraged to contact the research investigator for any queries about the study, using the provided contact information.

### Study participants

This cross-sectional study was used to assess the pattern and frequency of physical exercises and their effects on work-related musculoskeletal symptoms among clinical radiologists, including residents, specialists (junior staff), and consultants (staff) across all hospitals (academic, public, and private) in the major cities of the Eastern Province of Saudi Arabia.

### Recruitment of participants

We sent a personalized message with a link to the online survey to each of radiology residents practicing in the Eastern Province (*n* = 110) who were members of a WhatsApp (Facebook, Menlo Park, CA, USA) group. The link to the survey was also sent to radiology specialists and consultants whose contact information was available to the investigators. A reminder message was sent 3 days later.

Each invited radiologist received a unique link for the online survey so that the survey could not be filled more than once from the same link. This ensured that the survey would not be compromised by duplicate responses from the participants or responses from individuals not included in the target population. We used the QuestionPro respondent anonymity assurance feature to keep the identities of the participants anonymous.

Additionally, paper-based survey questionnaire forms were distributed to the radiology departments of hospitals in the surveyed region. They were intended for radiologists of whom the investigators did not have contact information. Investigators visited those departments a week later to collect the completed forms. The survey began on April 28, 2019 and lasted 14 days. Overall, the survey forms, whether online and paper-based, were distributed to a total of 263 radiologists.

### Structure of the questionnaire

This survey of the frequency and pattern of physical exercises was part of a series of surveys on the general health and wellbeing of radiologists in the Eastern Province of Saudi Arabia [19, 20]. The survey comprised 20 multiple-choice questions covering the following areas: (1) demographic information; (2) pattern and frequency of physical exercises; and (3) identification of work-related musculoskeletal symptoms.

The face and content validity of the survey questionnaire was verified by an expert panel of academicians. The relevance and appropriateness of each item were discussed. We conducted a pilot study with a group of 30 radiologists to assess the clarity of the questions and the time needed to complete the survey. After the pilot study, no major changes were made to the questions.

#### Exposure variables

The proposed risk factors were determined based on the literature focusing on demographic characteristics and work-related information. Demographic characteristics included age group, sex, years of practice, current institution of practice, and type of practice. Participants were asked how often they took part in different types of physical exercises (walking, running, group sports, swimming, stretching, strength training, functional fitness, and other aerobic exercises). A Likert-type scale (daily, weekly, monthly, less than monthly, and never) was used to record data about the frequency of physical exercises. In our present study, a physical exercise was defined to be more than 30 min.

#### Outcome variables

In this study, the outcome was the presence of work-related musculoskeletal symptoms which prevented the participants from doing normal physical activities within the last 12 months. These symptoms could be found in any of the nine body regions (i.e., neck, shoulder, elbow, wrist/hand, upper back, lower back, hip/thigh/buttock, knee, and ankle). The standard Nordic Musculoskeletal Questionnaire was used as it is a valid and reliable screening tool [21]. The responses of the outcome variables were dichotomized: responses of “left”, “right,” or “bilateral” in any body region were coded as a “yes”, whereas a respondent who indicated “no” for all body regions was coded as a “no”.

### Sample size estimation

The calculated sample size was 143 radiologists in order to detect an effect size of 0.3 with a two-sided alpha level below 0.05 and with a power of 80%. However, since the anticipated response rate was not expected to be high, the survey was distributed to almost twice the estimated sample size.

### Statistical analysis

The obtained data were compiled using the QuestionPro platform and analyzed using IBM SPSS for Windows, version 25 (IBM Corp., Armonk, NY, USA). Two questionnaire forms were excluded from the analysis because of missing data. All variables used in the study were categorical. Descriptive statistics, such as percentages and frequency distribution of different characteristics, were used as appropriate. For questions based on a Likert-type scale, five responses were dichotomized as “yes” and “no”. The Chi-square test was used to examine the bivariate relationship between the exposure and outcome variables. The level of statistical significance was set at *p* < 0.05.

## Results

### Characteristics of the participants

A total of 198 participants (an overall response rate of 75.3%), including residents (40.9%), specialists (27.3%), and consultants (31.8%), completed the survey. Besides, male participants outnumbered their female counterparts (56.1% vs. 43.9%). Most participants were in the 30–39 or <  30-year age groups, accounting for 40.4 and 30.8%, respectively. In addition, 36.4% of the participants had been working in the field of radiology for 1–5 years, whereas 33.9% had more than 10 years of work experience. Most participants (94.4%) were right-handed, and 57.6% reported spending 7 to 9 h daily at computer workstation interpreting and reporting radiological images (Table 1).

**Table 1:** Characteristics of the participants

Variable	N	(%)
**Age (years)**		
<30	61	(30.8)
30–39	80	(40.4)
40–49	35	(17.7)
50–59	15	(7.6)
≥60	7	(3.5)
**Gender**		
Male	111	(56.1)
Female	87	(43.9)
**Professional Rank**		
Resident	81	(40.9)
Senior Registrar	54	(27.3)
Consultant	63	(31.8)
**Years in Practice**		
<1	20	(10.1)
1–5	72	(36.4)
6–10	37	(19.7)
>10	67	(33.8)
**Institution**		
Academic	33	(16.7)
Public	124	(62.6)
Private	41	(20.7)

*N* number of respondents

### Physical exercises among the participants

#### Frequency of physical exercises

Seventy-five participants (37.9%) performed at least one type of physical exercises on a daily basis, compared to 55 (27.8%) and 23 (11.6%) on a weekly or monthly basis, respectively. Meanwhile, 45 participants (22.7%) did not regularly perform any physical exercises.

**Fig. 1:**
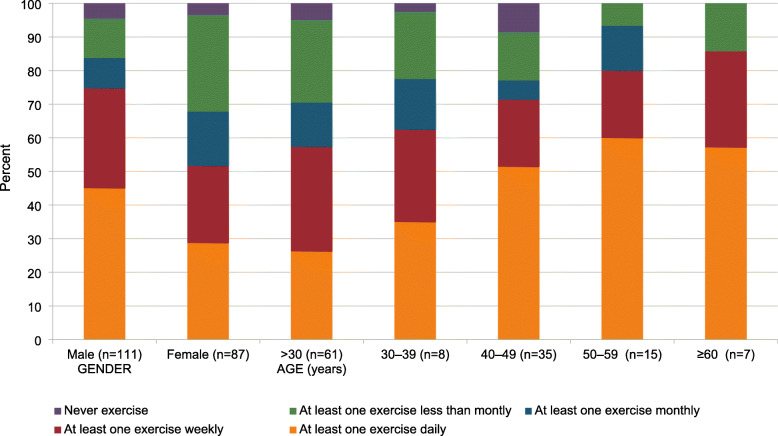
Frequency of physical exercises among genders and different age groups

There was a positive association, non-significant relationship between age and the frequency of physical exercises where older individuals were more likely to exercises frequently compared to younger individuals. Overall, 57.4, 62.5, 71.4, 80.0, and 85.7% of participants in the < 30-, 30-to 39-, 40-to 49-, 50-to 59-, and ≥ 60 -year age groups exercised at least once a week, respectively (Fig. 1).

Men were statistically more likely to exercise daily than women (*p* = 0.003). Overall, 83 male participants (74.8%) did a physical activity at least weekly, compared to 45 (51.7%) female participants. There were no statistically significant association between the frequency of physical exercises and the professional rank, the institution of practice, and the workload in terms of the time spent at a computer workstation reviewing medical images.

#### Pattern of physical exercises

The most frequently performed physical exercise was walking, as 138 participants (69.7%) reported walking regularly. Common physical exercises such as stretching, running, and other aerobic exercises were found in 81 (40.9%), 53 (26.8%), and 67 (33.8%) of the participants, respectively. However, group sports and swimming were the least popular type of physical exercise, reported by only 23 participants (11.6%).

**Fig. 2:**
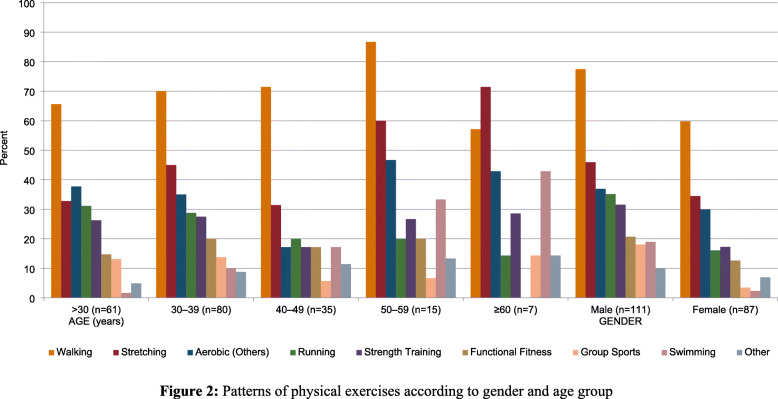
The pattern of physical exercises among genders and different age groups

Figure 2 demonstrates the patterns of physical exercises. The rank order of popular physical exercises was generally the same for age groups and genders. A notable exception is that stretching exercise is the most commonly found among individuals aged ≥60 years, compared to walking.

Age was significantly associated with swimming with older individuals being more likely to perform swimming than younger individuals (*P* = 0.004). For instance, 42.9% of participants aged 60 years and above do swimming regularly, compared to only 1.6% of those aged < 30 years. No similar significant associations were found between age groups and other physical exercises.

Gender was significantly associated with a number of physical exercises, including walking, running, group sports, swimming, and strength training (*p* < 0.05). Men were more likely to engage in these physical exercises than women (Table 2).

### Association between physical exercises and musculoskeletal symptoms

Overall, 176 (88.9%) participants reported having symptoms within the last 12 months prior to the study. These symptoms prevented 58 (29.3%) participants from doing usual physical activities due to these symptoms.

There was a statistically significant association between the frequency of physical exercises and the prevalence of disabling musculoskeletal symptoms within the 12 months prior to the survey. Overall, participants who did physical exercises regularly were less likely to have disabling musculoskeletal symptoms in any body region, including in the neck and shoulders. For instance, 60.9% of participants who did not do any physical exercise regularly reported having disabling neck pain. Meanwhile, such symptoms were lower among those who exercised monthly (45.8%) or at least weekly (32.8%) exercises. A similar pattern was observed when it came to shoulder pain. More specifically, 45.7% of participants who did not do physical exercises regularly suffered from disabling shoulder pain, whereas the figure for those who exercised at least once a week was only 25.8%.

**Table 2:** Frequency of physical exercise according to the participants’ characteristics

Variable	Total	At least one exercise weekly		At least one exercise monthly		Exercise less than monthly or never		*P*-value ^a^
		N	(%)	N	(%)	N	(%)	
**Age (years)**								
<30	61	35	(57.4)	8	(13.1)	18	(29.5)	0.479
30–39	80	50	(62.5)	12	(15.0)	18	(22.5)	
40–49	35	25	(71.4)	2	(5.7)	8	(22.9)	
50–59	15	12	(80.0)	2	(13.3)	1	(6.7)	
≥60	7	6	(85.7)	0	(0.0)	1	(14.3)	
**Gender**								
Male	111	83	(74.8)	10	(9.0)	18	(16.2)	**0.003**
Female	87	45	(51.7)	14	(16.1)	28	(32.2)	
**Years in Practice**								
<1	20	11	(55.0)	2	(10.0)	7	(35.0)	0.571
1–5	72	43	(59.7)	9	(12.5)	20	(27.8)	
6–10	39	26	(66.7)	6	(15.4)	7	(18.0)	
>10	67	48	(71.6)	7	(10.5)	12	(17.9)	
**Professional Rank**								
Resident	81	47	(58.0)	10	(12.4)	24	(29.6)	0.488
Senior Registrar	54	38	(70.4)	6	(11.1)	10	(18.5)	
Consultant	63	43	(68.3)	8	(12.7)	12	(19.1)	
**Overall**	198	128	(64.7)	25	(12.1)	46	(23.2)	

*N* number of respondents

^a^*P*-value in bold when significant

## Discussion

Regular physical exercise is probably the single most important intervention for preventing chronic diseases, making it one of the primary determinants of health [22]. The World Health Organization recommends at least 150 min of moderate-intensity physical exercises per week, i.e., 30 min a day, 5 days a week, for all adults [23]. Our study demonstrated that a high proportion (22.7%) of radiologists did not engage in regular physical activity and did not exercise even once a week. This is aggravated by the fact that the practice of clinical radiology has become more sedentary in the PACS era, potentially leading to negative effects on the general health of radiologists [18].

In our study, there was a significant difference in the frequency of physical exercise between men and women, with men being more likely to engage in most types of physical exercises. This might be due to traditional cultural practices and the differences in gender roles, which may contribute to the unavailability of exercise facilities and lead to time constraints due to child care requirements. Previous studies [24–26] have reported similar findings, with one study of 1360 adults in Malaysia demonstrating differences in the motives of exercise between men and women. According to this study, intrinsic factors (e.g., gaining strength) were more likely to motivate men to engage in physical exercise, whereas extrinsic factors (e.g., weight management and attaining an attractive appearance) were larger motivators among women [27].

Counterintuitively, our study found a positive association between age and the frequency of physical exercises. A possible explanation for this finding is the healthy worker effect phenomenon owing to which individuals who are physically inactive and have medical comorbidities are more likely to leave the workforce earlier. Unsurprisingly, walking was the most common physical exercise in our study, as it can be performed at any time and place.

Our findings demonstrated that participants performing regular physical exercises had a lower likelihood of experiencing disabling musculoskeletal symptoms, especially neck and shoulder pain, which is consistent with previous findings [28–30]. For example, a prospective study by Sitthipornvorakul et al. involving 387 workers revealed a negative association between the number of steps per day and the onset of neck pain. This study showed that for every 1000 extra steps walked in a day, the risk of neck pain decreased by 14% [29]. Moreover, in 2018, a systematic review of 11 studies conducted by Kelly et al. showed that exercise therapy is effective in reducing symptoms and improving function among sedentary workers with work-related upper limb disorders [30].

A large proportion of our study’s participants did not engage in regular physical exercises during their leisure time, indicating the importance of incorporating exercises into the hours spent at the workstation. In 2019, Johnson et al. conducted an interesting study to investigate the effects of treadmill workstation use on the interpretation of computed tomography findings by radiologists. Their study revealed that the use of such dynamic workstations did not significantly affect the detection of pulmonary nodules on CT scans, but shortened the duration required for interpretation [31]. Moreover, the use of standing radiology workstations is recommended to prevent the negative health effects of prolonged sitting time because it causes more energy expenditure than sitting [32–34]. To our knowledge, these radiology workstations are rarely used in the Eastern Province of Saudi Arabia. Therefore, given the magnitude of physical inactivity among radiologists, the use of such workstations should be promoted which may mitigate the health risks associated with sedentary work.

The present study has certain limitations. The frequency of physical exercises and the prevalence of work-related musculoskeletal symptoms were self-reported. Although self-reporting is time-saving and convenient, it may have led to some bias. In addition, the data on the frequency and duration of physical exercises collected in the study may not have been highly accurate, preventing the calculation of metabolic equivalent values, the preferred unit of measurement for physical activity. Finally, the cross-sectional nature of the study made it difficult to assess the causal relationship between physical inactivity and the occurrence of musculoskeletal symptoms.

## Conclusion

Our study showed that radiologists who performed regular physical exercises had a lower likelihood of experiencing work-related musculoskeletal symptoms. However, a significant number of radiologists, particularly those who were women, did not engage in regular exercises. Furthermore, interventional strategies aimed at promoting physical exercise among radiologists — such as gender-specific health promotion programs — should be developed.

## Data Availability

The datasets used and/or analyzed during the current study are available from the corresponding author on reasonable request.

## Abbreviations

CI: Confidence Interval
CT: Computed Tomography
OR: Odds Ratio
PACS: Picture Archiving and Communication Systems

## References

[1] Towbin AJ, Perry LA, Larson DB (2017). Improving efficiency in the radiology department. Pediatr Radiol.

[2] McEnery KW (2014). Coordinating patient care within radiology and across the enterprise. J Am Coll Radiol.

[3] Kyu HH, Bachman VF, Alexander LT, Mumford JE, Afshin A, Estep K, Veerman JL, Delwiche K, Iannarone ML, Moyer ML (2016). Physical activity and risk of breast cancer, colon cancer, diabetes, ischemic heart disease, and ischemic stroke events: systematic review and dose-response meta-analysis for the global burden of disease study 2013. BMJ.

[4] Michaud DS, Giovannucci E, Willett WC, Colditz GA, Stampfer MJ, Fuchs CS (2001). Physical activity, obesity, height, and the risk of pancreatic cancer. JAMA.

[5] Kushi LH, Doyle C, McCullough M, Rock CL, Demark-Wahnefried W, Bandera EV, Gapstur S, Patel AV, Andrews K, Gansler T (2012). American Cancer Society guidelines on nutrition and physical activity for cancer prevention: reducing the risk of cancer with healthy food choices and physical activity. CA Cancer J Clin.

[6] Proper KI, Singh AS, Van Mechelen W, Chinapaw MJ (2011). Sedentary behaviors and health outcomes among adults: a systematic review of prospective studies. Am J Prev Med.

[7] Dumith SC, Hallal PC, Reis RS, Kohl HW (2011). Worldwide prevalence of physical inactivity and its association with human development index in 76 countries. Prev Med.

[8] Ekelund U, Steene-Johannessen J, Brown WJ, Fagerland MW, Owen N, Powell KE, Bauman A, Lee IM (2016). Does physical activity attenuate, or even eliminate, the detrimental association of sitting time with mortality? A harmonised meta-analysis of data from more than 1 million men and women. Lancet.

[9] Matthews CE, Moore SC, Sampson J, Blair A, Xiao Q, Keadle SK, Hollenbeck A, Park Y (2015). Mortality benefits for replacing sitting time with different physical activities. Med Sci Sports Exerc.

[10] Stamatakis E, Gale J, Bauman A, Ekelund U, Hamer M, Ding D (2019). Sitting time, physical activity, and risk of mortality in adults. J Am Coll Cardiol.

[11] Vos T, Abajobir AA, Abate KH, Abbafati C, Abbas KM, Abd-Allah F, Abdulkader RS, Abdulle AM, Abebo TA, Abera SF (2017). Global, regional, and national incidence, prevalence, and years lived with disability for 328 diseases and injuries for 195 countries, 1990–2016: a systematic analysis for the global burden of disease study 2016. Lancet.

[12] Andersson HI (2004). The course of non-malignant chronic pain: a 12-year follow-up of a cohort from the general population. Eur J Pain.

[13] McBeth J, Silman AJ, Macfarlane GJ (2003). Association of widespread body pain with an increased risk of cancer and reduced cancer survival: a prospective, population-based study. Arthritis Rheum.

[14] Morken T, Magerøy N, Moen BE (2007). Physical activity is associated with a low prevalence of musculoskeletal disorders in the Royal Norwegian Navy: a cross sectional study. BMC Musculoskelet Disord.

[15] Nabeel I, Baker BA, McGrail JM, Flottemesch TJ (2007). Correlation between physical activity, fitness, and musculoskeletal injuries in police officers. Minn Med.

[16] Holth HS, Werpen HKB, Zwart J-A, Hagen K (2008). Physical inactivity is associated with chronic musculoskeletal complaints 11 years later: results from the Nord-Trøndelag health study. BMC Musculoskelet Disord.

[17] Balagué F, Bibbo E, Mélot C, Szpalski M, Gunzburg R, Keller TS (2010). The association between isoinertial trunk muscle performance and low back pain in male adolescents. Eur Spine J.

[18] Harisinghani MG, Blake MA, Saksena M, Hahn PF, Gervais D, Zalis M, da Silva Dias Fernandes L, Mueller PR (2004). Importance and effects of altered workplace ergonomics in modern radiology suites. Radiographics.

[19] Al Shammari M, Hassan A, Al Dandan O, Al Gadeeb M, Bubshait D: Musculoskeletal symptoms among radiologists in Saudi Arabia: a multi-center cross-sectional study. BMC Musculoskelet Disord 2019, 20(1):541. 10.1186/s12891-019-2933-1 PMC6857232 31727049

[20] Al Dandan O, Hassan A, Al Shammari M, Al Jawad M, Alsaif HS, Alarfaj K: Digital Eye Strain Among Radiologists: A Survey-based Cross-sectional Study. Acad Radiol 2020. 10.1016/j.acra.2020.05.006 32532637

[21] Crawford JO (2007). The Nordic musculoskeletal questionnaire. Occup Med.

[22] Ferguson B (2014). ACSM’s guidelines for exercise testing and prescription 9th Ed. 2014. J Can Chiropr Assoc.

[23] WHO. Global recommendations on physical activity for health. Geneva: World Health Organization; 2010. 26180873

[24] Cheah YK, Poh BK (2014). The determinants of participation in physical activity in Malaysia. Osong Public Health Res Perspect.

[25] Chen YJ, Huang YH, Lu FH, Wu JS, Lin LL, Chang CJ, Yang YC (2011). The correlates of leisure time physical activity among an adults population from southern Taiwan. BMC Public Health.

[26] Mao H-Y, Hsu H-C, Lee S-D (2020). Gender differences in related influential factors of regular exercise behavior among people in Taiwan in 2007: a cross-sectional study. PLoS One.

[27] Molanorouzi K, Khoo S, Morris T (2015). Motives for adult participation in physical activity: type of activity, age, and gender. BMC Public Health.

[28] Gerdle B, Brulin C, Elert J, Eliasson P, Granlund B (1995). Effect of a general fitness program on musculoskeletal symptoms, clinical status, physiological capacity, and perceived work environment among home care service personnel. J Occup Rehabil.

[29] Sitthipornvorakul E, Janwantanakul P, Lohsoonthorn V (2015). The effect of daily walking steps on preventing neck and low back pain in sedentary workers: a 1-year prospective cohort study. Eur Spine J.

[30] Kelly D, Shorthouse F, Roffi V, Tack C (2018). Exercise therapy and work-related musculoskeletal disorders in sedentary workers. Occup Med.

[31] Johnson CR, Besachio DA, Delonga D, Kuzniewski C, Mudge CS (2019). Effect of Dynamic Workstation Use on Radiologist Detection of Pulmonary Nodules on CT. J Am Coll Radiol.

[32] Hoffmann JC, Mittal S, Hoffmann CH, Fadl A, Baadh A, Katz DS, Flug J (2016). Combating the health risks of sedentary behavior in the contemporary radiology reading room. Am J Roentgenol.

[33] Levine JA, Schleusner SJ, Jensen MD (2000). Energy expenditure of nonexercise activity. Am J Clin Nutr.

[34] Richardson ML (2014). Wellness in the radiology Reading room: making your workstation a Workout Station. Am J Roentgenol.

